# Imprinted Gene Dosage Is Critical for the Transition to Independent Life

**DOI:** 10.1016/j.cmet.2012.01.006

**Published:** 2012-02-08

**Authors:** Marika Charalambous, Sacramento R. Ferron, Simao T. da Rocha, Andrew J. Murray, Timothy Rowland, Mitsuteru Ito, Karin Schuster-Gossler, Arturo Hernandez, Anne C. Ferguson-Smith

**Affiliations:** 1 Department of Physiology, Development and Neuroscience, University of Cambridge, Downing Street, Cambridge CB2 3EG, UK; 2 Institute for Molecular Biology, Medizinische Hochschule Hannover, Carl-Neuberg-Str, 1, D-30625 Hannover, Germany; 3 Department of Medicine, Dartmouth Medical School, Hanover, New Hampshire 03755, USA; 4 Department of Physiology, Dartmouth Medical School, Hanover, New Hampshire 03755, USA

## Abstract

Neonatal survival in mammals is crucially dependent upon maintenance of body temperature. Neonatal body temperature is largely maintained by thermogenesis in brown adipose tissue (BAT). BAT develops perinatally in mice requiring integration of adipogenic and thermoregulatory gene pathways. We describe a regulatory mutation in the imprinted gene cluster on mouse chromosome 12 resulting in early postnatal lethality. Maternal inheritance of this mutation impairs the ability of young mice to maintain body temperature. While mechanisms of perinatal BAT development are well understood, our work highlights a second phase of BAT recruitment necessary to support small animals newly independent of the nest. We show that the imprinted delta-like homolog 1/preadipocyte factor (*Dlk1/Pref1*) and iodothyronine deiodinase type 3 (*Dio3*) functions converge on the development of brown fat at the transition to independent life. This shows that appropriate dosage control at imprinted loci can act as a critical determinant in postnatal survival during phases of physiological adaptation.

## Graphical abstract

**Graphical Abstract:**

**Figure:**
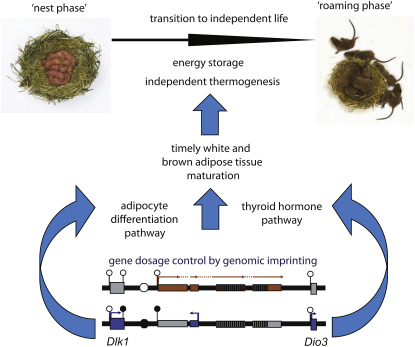

**Highlights:**

► Correct dosage of chromosome 12-imprinted genes is essential for postnatal survival ► Disruption of Dlk1 and Dio3 imprinting causes failure to adapt to independent life ► Maximal brown adipose activity is required as animals first leave the nest ► Dlk1 and Dio3 regulate maturation of brown adipose tissue

## Introduction

**Figure 1:**
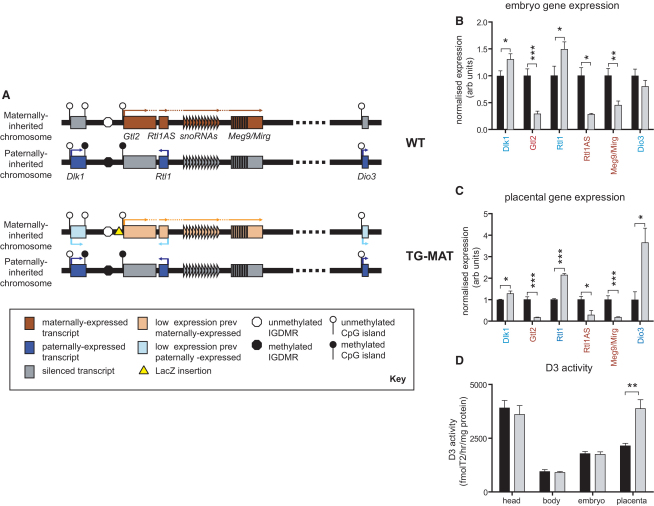
Disrupted Chromosome 12 Imprinting in TG^MAT^ Animals (A) Top: The maternally inherited chromosome is unmethylated at control regions and expresses a series of noncoding RNAs: *Gtl2*, *Rtl1AS*, *snoRNAs*, and *Meg9/Mirg* containing multiple microRNAs that initiate from the shared *Gtl2* promoter. *Dlk1*, *Rtl1*, and *Dio3* are silenced on this chromosome. The paternally inherited chromosome is hypermethylated, the noncoding RNAs are silenced, and protein-encoding genes are expressed. Bottom, summary of gene expression following maternal transmission of the Gtl2LacZ insertion. Protein-encoding gene silencing was partially alleviated, whereas noncoding RNA expression was reduced. The paternally inherited chromosome was not genetically modified. (B and C) Gene expression in the e16 embryo (B) and placenta (C), obtained by northern blotting (*Dlk1*), qRT-PCR (*Gtl2*, and *Meg9/Mirg*), and ribonuclease protection assay (RPA) (*Rtl1*, *Rtl1AS*, and *Dio3*). Data was normalized to WT = 1, n ≥ 6 conceptuses from at least 3 litters, ^∗^p < 0.05, ^∗∗^p < 0.01, ^∗∗∗^p < 0.001 by Mann-Whitney U test performed on prenormalized data. (D) D3 activity at e16. TG^MAT^ gray bars, WT black bars. All error bars represent SEM.

Imprinting (i.e., the differential expression of a gene depending upon its parental origin) arose in mammals coincidently with the evolution of homothermy and placentation (Edwards et al., 2007). Imprinted genes are commonly clustered, sharing regulatory elements (Ferguson-Smith, 2011). The *Dlk1-Dio3* region on mouse chromosome 12 contains paternally expressed protein encoding genes; *Dlk1*, *Rtl1* (retrotransposon-like gene) and *Dio3*; maternally expressed regulatory noncoding RNAs; *Gtl2*, *Rtl1AS* (a cluster of microRNAs that regulate *Rtl1 in trans*), *Meg8/snoRNAs*, and *Meg9/Mirg* (a large cluster of microRNAs whose targets have yet to be elucidated) (Figure 1A). Imprinting over the 1 Mb domain is controlled by an intergenic region that establishes differential DNA methylation in the male and female germlines (da Rocha et al., 2008).

The linkage of *Dlk1* and *Dio3* precedes the evolution of imprinting, and synteny is conserved in fish and birds (Edwards et al., 2008). This suggests that the genes are functionally linked (Fredman et al., 2009) and that selection has later acted to coordinately control their dosage by imprinting. However, a common process in which these genes act has not been described. Increased dosage of *Dlk1* both in vivo and in vitro in preadipocytes impairs their ability to differentiate into white adipose tissue (WAT) (Sul, 2009). *Dlk1* dosage in chondrocytes critically controls their maturation, suggesting that it may act more widely in the context of stem cell fate decisions (Sul, 2009). On the other hand, thyroid hormones (TH) control the rate of tissue maturation in early vertebrates and neuronal maturation in mammals (Williams, 2008). *Dio3* encodes the type 3 deiodinase (D3) that acts locally to reduce TH availability by inactivating both T3 and T4 (reviewed in Bianco et al., 2002). During mammalian intrauterine development, maternal circulating TH far exceeds that seen by the fetus due to the action of D3 at the placental interface (Bates et al., 1999). Deletion of *Dio3* causes perinatal thyrotoxicosis and persistent changes to the TH axis by both central mechanisms and also by impairing thyroid gland development (Hernandez et al., 2006, 2007). A recent study demonstrated that both *Dlk1* and *Dio3* expression was elevated in cultured brown preadipocytes and downregulated during differentiation, suggesting that imprinting might control the dosage of these genes to regulate thermogenesis (Hernandez et al., 2007). However, very little D3 is expressed in vivo in brown adipose tissue (BAT) and the significance of *Dio3* in this cell culture model is unclear. Moreover, a role for *Dlk1* in preweaning BAT differentiation in vivo has not been established.

Between postnatal days 14 and 21 (P14–P21), mice have opened eyes, fur, and the capacity for independent feeding (Rugh, 1968). The transition to independent life places new demands upon the animal. First, weaning comprises a dietary switch from the almost continuous infusion of fatty acid-rich milk to predominantly carbohydrate-containing solid food, ingested at intervals. Therefore, in this roaming phase the animal must be capable of digesting solid food and storing excess energy as fat, thus shifting their metabolic mode from lipolytic to lipogenic (Herrera and Amusquivar, 2000). Second, the animal must control its own appetite and long-term energy homeostasis. Third, it must be able to maintain its body temperature away from the nest. These requirements are associated with dynamic alterations in hormone levels in the preweaning period, such as growth hormone (Lupu et al., 2001), corticosterone (Henning, 1978), leptin (Ahima et al., 1998), and thyroid hormones (Hernandez et al., 2006), which drive growth and prepare the future metabolic organs and hypothalamic-pituitary-adrenal (HPA) axis for independent life (Fowden et al., 1998). The crucial role of leptin in modulating hypothalamic circuitry is well established (Bouret et al., 2004; Pinto et al., 2004) but the molecular events necessary for in vivo maturation of adipose tissue are not well understood.

Timely WAT maturation is necessary for both energy storage and for the production of leptin (Ahima et al., 1998), whereas adequate BAT recruitment is necessary to support thermogenesis in small animals newly independent of the nest. During the preweaning period, the small size of juvenile mice makes them acutely sensitive to cold, and during this period they can be considered to be under constant cold stress. In the postnatal period, rodents generate most of their body heat by nonshivering thermogenesis (NST). NST is performed in BAT by the uncoupling protein UCP1, which diverts cellular respiration from chemical energy production into heat generation (Cannon and Nedergaard, 2004). Brown adipose deposits can be detected in the rodent embryo from late gestation but thermogenic capacity increases during the first week of life, requiring the concerted actions of adipogenic factors, sympathetic stimulation, and thyroid hormones (Giralt et al., 1990).

We have previously described several genetic models that result in the complete loss of imprinting at mouse chromosome 12 (da Rocha et al., 2008), such that *Dlk1*, *Rtl1* and *Dio3* are fully expressed from both parental alleles and noncoding RNA expression was repressed. These mutants die before birth with defects in placentation and organ maturation, demonstrating the crucial role of imprinting in embryonic development (da Rocha et al., 2008). To investigate the role of chromosome 12 imprinted genes in postnatal life, we utilized Gtl2LacZ transgenic mice (Schuster-Gossler et al., 1996). This genetic model results from the insertion of 2–3 copies of a promoterless βgeo cassette 1.7 kb upstream of the *Gtl2* promoter (Paulsen et al., 2001). This partial loss of imprinting results in alteration of transcript dosage of chromosome 12-imprinted genes, and the mice are viable and fertile (Schuster-Gossler et al., 1998; Sekita et al., 2006; Steshina et al., 2006).

We found that maternal transmission Gtl2LacZ transgenic animals showed significant lethality in the third postnatal week, associated with developmental delay and failure to coordinate essential adaptations to independent life and culminating in the failure to maintain UCP1 expression in BAT. This defect was the combined result of prolonged elevated expression of *Dlk1*, causing a failure of BAT differentiation and consequently reducing expression of β-adrenergic receptors, and hypothyroidism due to misregulation of *Dio3*. Our results show that small coordinated alterations in imprinted gene dosage in the context of a cluster have a major impact on postnatal survival. These findings link the underlying mechanism of dosage control by imprinting with critical physiological determinants to maintain imprinted gene clustering throughout mammalian evolution.

## Results

### Maternal Transmission of the Gtl2LacZ Insertion Results in Disrupted Chromosome 12 Imprinting without Embryonic Lethality

We evaluated gene expression in the wild-type (WT) and mutant late gestation embryo and placenta, when gene expression is maximal. Consistent with previous reports (Sekita et al., 2006; Steshina et al., 2006), we found that maternal transmission Gtl2LacZ transgenic embryos (hereafter TG^MAT^) exhibited partial paternalization of the maternally inherited chromosome (summarized in Figure 1A). Thus, *Dlk1* and *Rtl1* expression was increased by 30%–50%, whereas noncoding RNA (*Rtl1AS*, *Gtl2*, and *Meg9/Mirg*) expression was substantially reduced (Figure 1B). In the placenta, *Dio3* as well as *Dlk1* and *Rtl1* had significantly elevated gene expression (Figure 1C). DLK1 protein levels were increased in the mutant embryo and placenta in a spatial pattern that was indistinguishable from that of WT littermates (Figure S1 available online). The enzymatic activity (D3) of the type 3 deiodinase was increased in the TG^MAT^ placenta by approximately 2-fold (Figure 1D). In common with paternal disomy for this region (PatDpDi12), TG^MAT^ conceptuses in late gestation had large placentae (Figure S1C). However, unlike PatDpDi12 conceptuses, TG^MAT^ embryos reached late gestation with no overt phenotypic differences and at expected Mendelian frequencies (Figure S1C).

### Impaired Postnatal Survival in TG^MAT^ Neonates Is Biphasic and Associated with Failure to Thrive

**Figure 2:**
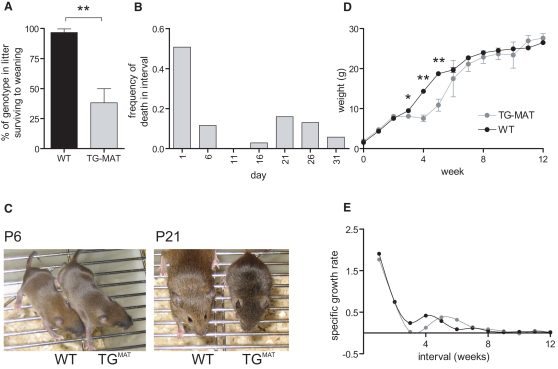
Impaired Postnatal Survival in TG^MAT^ Neonates Is Biphasic and Associated with Failure to Thrive (A) Percentage of animals surviving to weaning in 11 litters generated by maternal transmission of the Gtl2LacZ transgene, ^∗∗^p < 0.01, Mann-Whitney test. (B) Day of death was ascertained for 69 TG^MAT^ animals and is presented as frequency of animals dead during a 5-day interval. (C) Appearance of WT and mutant littermates at P6 and P21. (D) TG^MAT^ animals (all surviving for 12 weeks, males; n = 8) and 30 WT littermates (n = 30) were weighed weekly for 12 weeks. ^∗^p < 0.05, p < 0.01 by one-way ANOVA with Bonferroni's multiple comparison post tests; error bars represent SEM. (E) Growth rates derived from the data in D, calculated using the equation: (weight at T_2_ − weight at T_1_)/ weight at T_1_.

After birth, only 38% of TG^MAT^ animals survived to weaning at 4 weeks (42 out of 111 mice; Figure 2A). Of those not surviving to weaning, 32% died within the first few days of birth with respiratory problems (35 out of 111 mice; Figures 2B and S2). Importantly, TG^MAT^ mice surviving to P6 were indistinguishable from WT littermates, but at P21 mutant animals were smaller and appeared developmentally delayed (Figure 2C). About half of these remaining animals died before weaning at P28 (34 out of 111 mice; Figure 2B). This second phase of mortality was preceded by a period of developmental delay, commencing at ∼P14, during which TG^MAT^ pups failed to gain weight (Figure 2D). The surviving animals resumed a normal growth trajectory after the crisis at week 2–3, as illustrated by the specific growth rate curve for TG^MAT^ pups, which has a similar shape to the WT but shifts to the right (Figure 2E). Events leading to this second phase of mortality are the primary focus of this study.

### Juvenile TG^MAT^ Animals Feed Normally but Fail to Adjust to a High Carbohydrate Diet and to Accumulate Fat Stores

Leaving the nest and entering the roaming phase is associated with changes in the metabolic mode from an early lipolytic state, where growth and peripheral metabolism are mostly fuelled by lipid, to a lipogenic state, where carbohydrates are the major fuel (Herrera and Amusquivar, 2000). At P6, WT pups have high circulating triglycerides (TAG, Figure S3A), which decline ∼10-fold between P14 and P21, corresponding to the interval when young animals begin to supplement their milk diet with solid food. During this period pups also begin to accumulate abdominal WAT. At P11, < 10% of WT animals had visible abdominal WAT deposits; by P14, 25% had gained WAT and by P21, > 70% had done so (Figure S3B). TG^MAT^ animals experienced a developmental delay in the transition from a lipolytic to a lipogenic mode. Serum TAG remained relatively high at P11 and animals failed to accumulate abdominal WAT at the same rate as their WT littermates (Figures S3A and S3B). Of the small number of mutant animals that accrued WAT at P14, the TAG content within this tissue was reduced (Figure S3A). In contrast TAG accumulated in the muscle and liver of mutant animals between P11 and P14, indicating a defect in adipose fat storage (Figure S3A). During the same period, free-fed stomach weights were not reduced, indicating that there was no primary defect in feeding, and free-fed insulin levels were normal (Figure S3A and Tables S1 and S2). Consistent with its established role in adipogenesis (Sul, 2009), we suspected that this primary defect in WAT was caused by overexpression of *Dlk1.* We were unable to establish a role for local DLK1, due to the small amount of WAT material available from TG^MAT^ animals at P11. However, inhibition of adipogenesis has also been associated with elevated levels of circulating DLK1 (Sul, 2009). Whereas circulating DLK1 decreased ∼ 5-fold in WT animals, this decrease was delayed in the mutants (Figure S3A).

The increase in fat storage during the roaming phase is associated with a shift in the expression of hepatic genes. In WT mice, expression of the lipogenic genes *Scd1* and *Spot14* was low at P11, whereas lipolytic *Pparα* was high (Figure S3C). In contrast, expression of hepatic *Scd1* and *Spot14* expression was high by P21 and *Pparα* was reduced. The livers of TG^MAT^ mice had altered hepatic gene expression. While normal levels of *Pparα* and *Pepck* indicated that lipolytic and gluconeogenic pathways were not affected, lipogenic *Spot14* and *Scd1* gene expression was reduced (Figure S3C). We could not detect *Dio3* mRNA in the liver at P11 or P21 (data not shown), and local *Dlk1* expression was low and did not differ between WT and mutants (Figure S3C), suggesting that the metabolic defects in the liver could have an endocrine cause.

### Persistent Gestational Hypothyroidism in TG^MAT^ Juveniles

**Figure 3:**
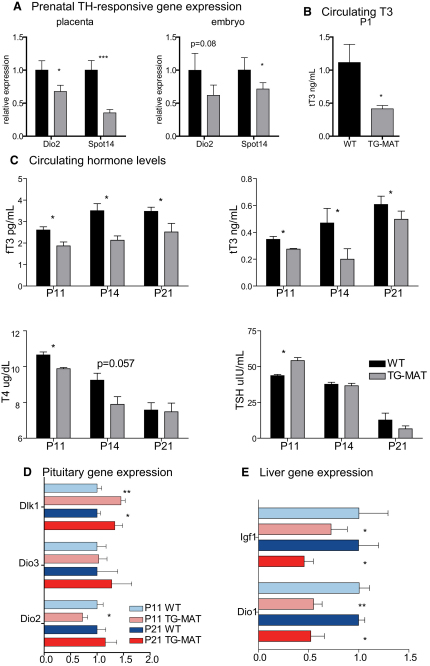
Juvenile TG^MAT^ Animals Are Hypothyroid (A) Expression of thyroid hormone responsive genes in the e16 embryo and placenta assessed by qRT-PCR as described above. (B) Serum total T3 at P1 (n = 6/genotype). (C) Measurement of serum thyroid hormones of TG^MAT^ animals with WT littermates at P11 (n = 6/genotype), P14 (n ≥ 4/genotype) and P21 (n ≥ 10/genotype). Because sexual dimorphism was not observed in either body weight or serum parameters at any stage, the data was combined. (D) cDNA was generated from individual pituitaries at P11 (n = 6/genotype), and at P21 (n ≥ 11/genotype), and gene expression quantified by qRT-PCR normalized to Hprt and represented as relative to WT = 1. (E) qRT-PCR analysis of preweaning liver gene expression. cDNA was generated from individual livers at P11 and at P21 (n = 9/stage/genotype) and data was analyzed as described in (B). *Spot14* could not be detected at P11. All comparisons are by Mann-Whitney U test; ^∗^p < 0.05, ^∗∗^p < 0.01, ^∗∗∗^p < 0.001. Error bars represent SEM.

Impaired thyroid signaling is associated with phenotypic consequences, including impaired hepatic lipogenesis downstream of *Spot14*, growth retardation caused by reduced IGF1 production, and developmental delay of several organs, including the gut (Fraichard et al., 1997). We observed that TG^MAT^ placentae exhibited elevated D3 activity during late gestation (Figure 1D). Because placental D3 acts as a barrier to maternal thyroid hormones (Bates et al., 1999), we wished to determine whether TG^MAT^ conceptuses were gestationally hypothyroid. To do this, we measured the expression of genes that are known to be responsive to thyroid hormone signaling. Expression of *Dio2* and *Spot14* was significantly reduced in the placenta at e16, (Figure 3A), consistent with placental hypothyroidism. Spot14 gene expression was also reduced in the embryo, indicating that hypothyroidism occurs even in the absence of locally elevated D3. Because circulating T3 was also reduced on the day of birth, gestational hypothyroidism persisted postnatally in TG^MAT^ animals, (Figure 3B).

To test whether TG^MAT^ animals were also hypothyroid during the critical roaming phase, we measured circulating free and total T3, total T4, and thyroid-stimulating hormone (TSH) levels before the failure to thrive (P11) at the onset of growth retardation (P14) and in surviving animals at P21. In the early postnatal period, T3 and T4 levels were low in mutants and circulating TSH was significantly elevated but failed to normalize T4 (Figure 3C). We found evidence of a defect in TH feedback mechanisms at P11 because deiodinase 2 (*Dio2*) expression was reduced in the pituitary. A role for pituitary *Dio3* at this time can be ruled out because levels were very low and did not differ between mutants and WT littermates. *Dlk1* was abundantly expressed in the pituitary at this stage, with mutant animals exhibiting *Dlk1* overexpression (Figure 3D).

**Figure 4:**
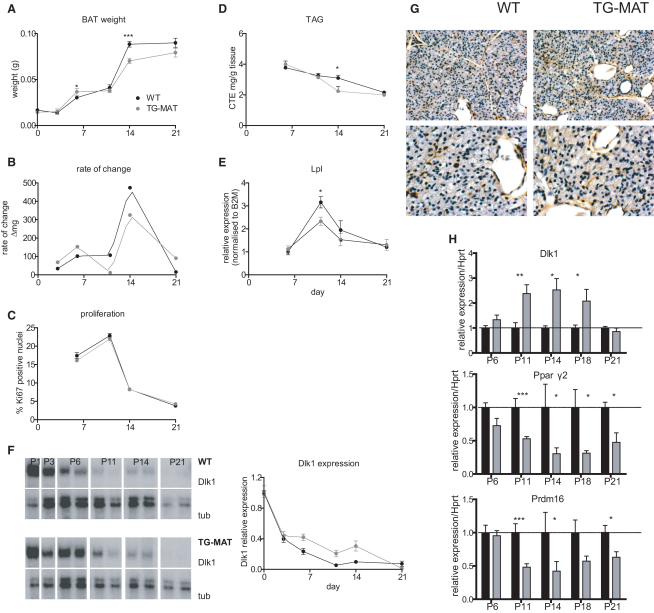
BAT Recruitment Dynamics Are Altered in TG^MAT^ Animals (A) BAT weight at P0 to P21 (see Table S1 for statistics). (B) The rate of change of BAT weight over 21 days, derived from the data in (A). (C) Proliferation of BAT at P6 to P21 measured using number of cells staining positive for Ki67, n = 4–5 individuals/stage/genotype. (D) Tissue TAG between P6 and P21, expressed as mg triolein units/g tissue, n = 6–16 samples/stage/genotype, ^∗^p < 0.05 Mann-Whitney U test. (E) qRT-PCR was performed on BAT at P6 (WT n = 9; TG^MAT^ n = 11), P11 (WT and TG^MAT^ n = 6), P14 (WT and TG^MAT^ n = 6) and P21 (WT n = 8; TG^MAT^ n = 12). All developmental stages were performed on the same plate and normalized to a single standard curve, then normalized to WT P6 = 1. Prenormalization values were compared using a Mann-Whitney U test, ^∗^p < 0.05. (F) Quantification of *Dlk1* mRNA expression over the first three weeks of life by RNase protection assay (autoradiograph, left panel, and quantification graph, right panel, n = 4 animals/stage, below). (G) Anti-DLK1 immunohistochemistry in P11 BAT (top panels = 200× magnification; bottom panels = 400×). (H) qRT-PCR performed on BAT at P6–P21 as in (E) but including P18 (WT n = 7; TG^MAT^ n = 8). Data was normalized to WT = 1. Prenormalization values were compared using a Mann-Whitney U test; ^∗^p < 0.05, ^∗∗^p < 0.01, ^∗∗∗^p < 0.001. All error bars represent SEM.

Interestingly, in surviving animals at P21, serum T4, TSH, and pituitary mRNAs for *Dio2* had normalized but T3 levels remained low. This suggests increased clearance of T3 to T2 by D3 and/or reduced generation of T3 from T4 by deiodinase 1 or 2 (D1 or D2). Normally, D3 activity decreases postnatally in most tissues but remains high in the central nervous system (Bates et al., 1999). To determine a potential role for D3 in this process, we measured D3 activity in BAT and whole brains of WT animals but could detect no differences in D3 activity in either tissue in WT and TG^MAT^ animals at P14 or at P21 (Figure S3A). Therefore, we postulated that there might be a defect in T4 to T3 deiodination that could cause the reduction in circulating T3 in the mutants. Consistent with this, we found reduced hepatic expression of *Dio1* mRNA as well as lower levels of mRNA for the TH-responsive *Spot14* and *Igf1* genes (Figures 3C and 4E). Therefore, the hypothyroid state of the TG^MAT^ liver likely maintains gestational low T3 by reducing T4 deiodination in a feedback mechanism because hepatic *Dio1* itself is positively regulated by thyroid hormones (Gereben et al., 2008).

TG^MAT^ animals are growth-retarded (Figure 2C), consistent with reduced hepatic *Igf1* mRNA (Figure 3E), and mutants also had reduced circulating IGF1 at P21 (Figure S4C). We investigated whether gut development was affected in mutants by measuring indices of maturation, such as villous size and number as well as muscle cross sectional area. The jejunum (and to a lesser degree the ileum) of TG^MAT^ displayed reduced villous height, diameter, and number and a reduction in the cross-sectional area of the longitudinal and transverse muscle (Figure S4B), impairments that are consistent with developmental delay (Fraichard et al., 1997).

### A Previously Undescribed Second Phase of BAT Recruitment Is Reliant on Dlk1 Dosage

The hunched, piloerect appearance of TG^MAT^ animals (Figure 2D) suggested that they might have a defect in thermoregulation. Previous studies have described the successive recruitment of BAT during the first five postnatal days (summarized in Cannon and Nedergaard, 2004). TG^MAT^ animals had slightly increased BAT weight during this period (Figure 4A and Table S1).

Between P11 and P21, we observed that WT mice experience a second phase of BAT recruitment, during which the interscapular brown adipose compartment reaches adult size (Figures 4A and 4B). This corresponds to the period when animals begin to leave the nest and the maintenance of body temperature is less reliant on the mother and more dependent on the individual, although body size is still small. The increase in BAT size at this time is likely due to increased cell number because proliferation peaks during this period (Figure 4C), but TAG accumulation is fairly stable (Figure 4D). However, lipoprotein lipase (*Lpl*) expression is sharply upregulated between P11 and P11 in WT animals. Because LPL figures importantly in releasing fatty acids from circulating TAG into cells, suggesting that BAT's use of fatty acids as fuel is likely to be maximal (Figure 4E).

The second phase of BAT recruitment was impaired in TG^MAT^ animals (Figures 4A and 4B). Specifically, mutant animals failed to develop BAT levels appropriate to body weight at P14 (Table S1). *Dlk1* is expressed in preadipocytes of both brown and white fat (Sul, 2009; Tseng et al., 2005). In WT BAT, *Dlk1* expression sharply declined in the first 10 days of life; however, this decline was delayed in TG^MAT^ animals, resulting in elevated *Dlk1* mRNA at the time of failed BAT recruitment (P6 to P18, Figure 4F). During this period, we could detect neither *Rtl1* nor *Rtl1AS* transcripts and although *Gtl2* tended to be reduced in mutants (and also declined by P21), it was very variable and expression was not significantly altered in TG^MAT^ animals (data not shown). Hence, this BAT phenotype can be attributed to failure to downregulate *Dlk1*.

Because we observed no differences in the rate of proliferation between the two genotypes (Figure 4C), we hypothesized that elevated *Dlk1* might impair the second wave of BAT recruitment by preventing preadipocyte differentiation consistent with its established role in WAT (Sul, 2009). In P11 BAT, *Dlk1* was expressed in the vascular and mesenchymal cells and in a subset of adipocytes, and *Dlk1* positive cells appeared to comprise a greater proportion of the BAT compartment in TG^MAT^ animals (Figure 4G). In mutants, TAG accumulation was impaired and *Lpl* expression was reduced (Figures 4D and 4E). Finally, TG^MAT^ animals had reduced expression of differentiated adipogenic markers, such as *Pparγ2* and *Prdm16*, supporting the observation that, from P11, mutant BAT is retarded in an immature state associated with *Dlk1* overdose (Figure 4H).

### Reduced Expression of Adrenoreceptors Provides Further Evidence of Developmental Delay in TG^MAT^ BAT

**Figure 5:**
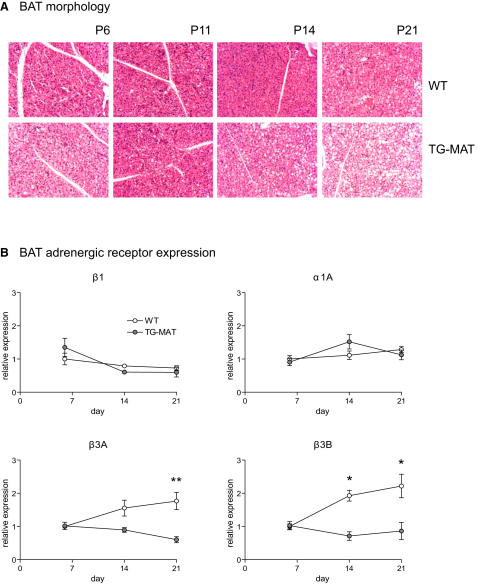
Developmental Induction of β3 Adrenoreceptors Is Not Observed in TG^MAT^ Juveniles (A) Morphology of WT and TG^MAT^ BAT from P6–P21. H&E staining at 200 × magnification. Note large white circular fat deposits in the mutant cells at P14 and P21. (B) qRT-PCR performed on BAT at P6–P21 (n > 4/stage/genotype), all performed on the same plate and normalized to a single standard curve, then normalized to WT P6 = 1. Prenormalization values were compared by Mann-Whitney U test; ^∗^p < 0.05, ^∗∗^p < 0.01. All error bars represent SEM.

Histological examination of BAT at P21 revealed impaired differentiation of the mature adipocytes. TG^MAT^ cells accumulated fat in large central vesicles reminiscent of WAT (Figure 5A) and similar to that observed in the BAT of mice with impairments in the β-adrenergic pathway (Thomas and Palmiter, 1997). Sympathetic nervous system (SNS)-derived norepinephrine (NE) acting on BAT adrenoreceptors has multiple effects upon BAT recruitment, and the receptors are dynamically regulated (summarized in Collins et al., 2004). Cultured brown preadipocytes express the β1-adrenergic receptor (AR), but the β3-ARs become most abundant during differentiation to mature adipocytes (Bronnikov et al., 1999). In normal individuals, we observed an increase in expression of β3-ARs between P6 and P21 (Figure 5B), consistent with our prediction that in vivo BAT terminal differentiation occurs during this period. We found no change in the expression of the *β1-AR* and only a modest increase in *α1-AR* expression over time, as expected from in vitro data (Bronnikov et al., 1999; Granneman et al., 1997). Importantly and in contrast to WT juveniles, TG^MAT^ animals did not induce expression of *β3-AR*. This is consistent with the immaturity of the brown adipocytes during this period. The proliferative effect of NE is thought to be mediated by the β1-AR in preadipocytes (Bronnikov et al., 1999). We saw no reduction in *β1-AR* expression in TG^MAT^ animals, consistent with their normal BAT proliferation (Figure 4D). Expression of the *α1A-AR*, which augments the action of the β3-ARs, was not altered in the mutants. Because the transcriptional effects of NE on activation of thermogenic genes in BAT are mediated predominantly via β3-ARs (Bronnikov et al., 1999), we postulated that reduced expression of β3 adrenoreceptor in the mutants might lead to defects in the expression of these genes.

### BAT Thermogenesis Is Impaired in TG^MAT^ Juveniles

**Figure 6:**
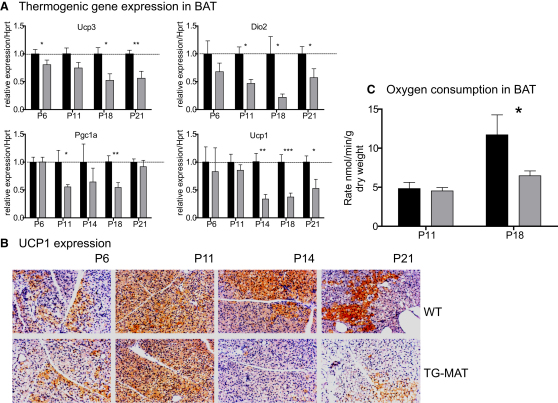
Thermogenic Properties of BAT in the Preweaning Period (A) qRT-PCR performed on BAT at P6–P21 as in Figure 5H. Data was normalized to WT = 1. Prenormalization values were compared by Mann-Whitney U test; ^∗^p < 0.05, ^∗∗^p < 0.01, ^∗∗∗^p < 0.001. (B) UCP1 expression in BAT at P6–P21. UCP1-positive cells are stained in brown and cells were counterstained with hematoxylin (blue). (C) Oxygen consumption measured in BAT explants in the presence of NE (1 μM) and succinate (10 mM), normalized to tissue dry weight. P11 WT n = 5, TG^MAT^ n = 6, P18 WT n = 6, TG^MAT^ n = 10; ^∗^p < 0.05 Mann-Whitney U test. All error bars represent SEM.

β3-AR stimulation results in an increase in intracellular cAMP, which activates the transcription factor CREB. CREB targets in BAT include *Ucp1* and *Dio2*, as well as *Pgc1α*, the master regulator of mitochondrial biogenesis ([Silva and Larsen, 1986] and reviewed in [Collins et al., 2004]). We found that TG^MAT^ animals had reduced expression of these genes during the critical period (Figure 6A).

Thyroid hormones augment the transcriptional activation of *Dio2* (Martinez-deMena et al., 2002) and *Ucp1* (Bianco and Silva, 1987) by β-adrenergic signaling. Consistent with the underlying hypothyroid state (Gong et al., 1997), we found that, from P6, TG^MAT^ animals had reduced expression of *Ucp3* in BAT (Figure 6A). *Dio2* mRNA was also reduced at this stage in mutants (i.e., prior to the predicted reduction in β3-AR stimulation) (Figure 5B), suggesting an involvement of low T3. Expression levels of thermogenic genes were highly variable, perhaps reflecting the spectrum of phenotypic outcomes. Crucially, the expression of *Ucp1* was reduced at P14, P18, and P21 (Figure 6A).

Immunohistochemical staining for UCP1 in BAT confirmed our earlier prediction that thermogenesis at P11 is induced to a high level because most BAT cells appeared densely stained at this time (Figure 6B). Widespread UCP1 expression is maintained at P14 in WT animals but declines to a pattern of punctate staining by P21. In the mutant, high UCP1 is induced at P11 but not maintained, and expression declines at P14 and P21. Correspondingly, the maximal rate of oxygen consumption per unit dry weight in the presence of NE was the same in BAT explants from WT and TG^MAT^ animals at P11 but increased only in WT animals by P18, such that oxygen consumption was ∼2 fold lower in TG^MAT^ animals during the roaming stage (Figure 6C).

### TG^MAT^ Juveniles Fail to Maintain Body Temperature away from the Nest and Survive at Thermoneutrality

**Figure 7:**
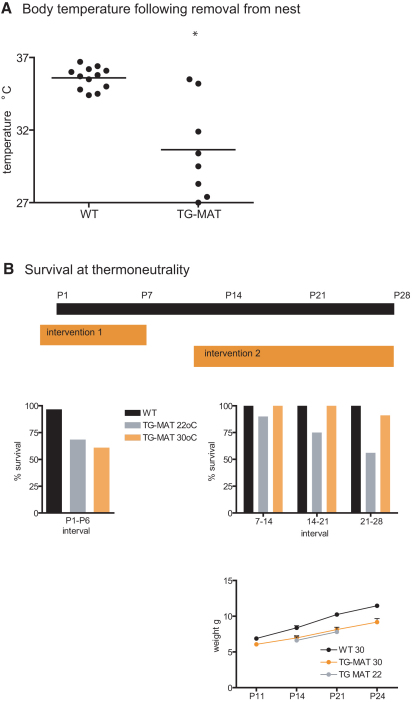
Cold Challenge and Rescue at Thermoneutrality (A) Rectal temperature of individual animals at P18 following removal from the nest for 2 hr. WT n = 12, TG^MAT^ n = 8 (3 litters); ^∗^p < 0.05 Mann-Whitney U test. (B) Top, schematic of experiments conducted to investigate the effect of ambient temperature on mortality. In intervention 1 (left graph), mothers were placed at 30°C 2 days prior to parturition and maintained at 30°C until P6 or at room temperature (22°C) during this period. Pups were counted daily and % survival was calculated. Of 37 animals born at 30°C, 22 survived; of 111 animals born at 22°C, 76 survived. In intervention 2 (right graph, top), pups were raised at room temperature until P11, then placed at 30°C or maintained at 22°C until P28. Of the 76 animals incubated at room temperature between P11 and P28, 34 survived, whereas of the 33 animals incubated at 30°C in this interval, 30 survived. During both interventions, the nest was kept intact and the mother was not removed. Bottom right, body weights of animals raised at room temperature or at 30°C between postnatal day 11 and 24. Error bars represent SEM.

We next tested the ability of TG^MAT^ animals to defend their body temperature during the critical phase by removing them from the nest for 2 hr. Following this challenge, the median rectal temperature of WT animals was 35.8°C, with very little variation (interquartile range 34.9°C–36.2°C), whereas the mutants had a median temperature of 30.0°C, with a large variation (27.9°C–33.6°C) (Figure 7A), indicating that some mutant animals were experiencing hypothermia.

To determine whether failure to defend body temperature was the cause of death in TG^MAT^ animals, we attempted to rescue animals at both phases of mortality by raising them at thermoneutrality (Figure 7B). For the first intervention, we compared mutant animals raised at 22°C (normal housing conditions) with those whose mothers were placed at 30°C 2 days prior to parturition. At P6 we observed that neonatal mortality was not rescued by our intervention (61% of TG^MAT^ pups survived at 30°C compared to 68% at 22°C). For the second intervention, we raised nine litters at 30°C from P11. Under these conditions, 91% of TG^MAT^ animals were still alive at P28 (30/33) in contrast to 56% of TG^MAT^ animals that survived the same interval under normal housing conditions (43/77, Figure 7B). However, we found that the body weight phenotype of the mutant animals was not rescued by increasing the ambient temperature, suggesting that mortality was not secondary to growth retardation. We therefore concluded that hypothermia was the most likely cause of death of TG^MAT^ animals.

## Discussion

We have utilized mice with a regulatory mutation at the imprinted domain on chromosome 12 to demonstrate that even a modest perturbation of gene dosage in the context of an evolutionarily conserved gene cluster can result in a dramatic reduction in postnatal fitness. Other genetic models that paternalize the maternally inherited copy of chromosome 12 result in late embryonic lethality (da Rocha et al., 2008); however, the partial paternalization of gene expression caused by insertion of a transgene onto the maternally inherited chromosome was not lethal during gestation. TG^MAT^ embryos appeared normal, though their placentae were overgrown by 10%–20%. This is likely due to increased *Rtl1* dosage because animals overexpressing this gene in a genetic model that ablates an inhibitory transcript, *Rtl1AS*, have a similar phenotype with no apparent effect of increased *Rtl1* gene dosage on the fetus (Sekita et al., 2008). We have previously reported that complete paternalization of chromosome 12 in uniparental disomy conceptuses results in fetal hypothyroidism due to *Dio3* overexpression (Tsai et al., 2002). Placental D3 acts as a barrier to maternal thyroid hormones, and fetal production of these hormones initiates late in the gestation in rodents (Bates et al., 1999). We observed in mutants that *Dio3* mRNA and enzyme activity was elevated in the late-gestation placenta, causing hypothyroidism of the TG^MAT^ embryo and placenta that persisted at birth.

TH levels peak in rodents between P10 and P14 (Hernandez et al., 2006), the time at which TRα signaling is transiently required for the transition to independent life (Fraichard et al., 1997). We found that TG^MAT^ pups had a complex misregulation of thyroid hormone homeostasis, an early period of central hypothyroidism followed by defects in peripheral conversion of T4 to T3. We found that early hypothyroidism (P11) was due to a central defect in TH production that could not be explained by an increase in central D3 at this stage, although it might be a consequence of gestational hypothyroidism.

Circulating T3 is maintained by the action of deiodinases D1, D2 and D3. At P21, we observed that *Dio1* expression and that of other TH-responsive genes was reduced in the liver. In addition, *Dio2* expression in BAT was reduced. Therefore, low serum thyroid hormones in TG^MAT^ animals may be explained by a combination of reduced hepatic *Dio1* and low *Dio2* expression in BAT (Figures 3E and 6A). The inadequate response of pituitary TSH may reflect a lag time required by the pituitary to reset after alteration of the thyroid hormone load. It is also possible that some alterations of the TH axis in TG^MAT^ animals are an indirect effect of reduced fat storage and serum adipokine concentrations (e.g., leptin, Table S1).

Between postnatal days 14 and 21 (P14–P21), mice leave the nest and commence independent feeding and temperature regulation. TG^MAT^ animals have poor survival at this transition to independent life. First, TG^MAT^ pups fail to adapt to the dietary switch from fatty acid-rich milk to carbohydrate-rich chow. We found that mutant animals displayed reduced expression of hepatic genes in the lipogenic pathway, at least partly due to the hypothyroid state of the liver. Moreover, we observed developmental delay of the small intestine and reduced growth associated with low circulating IGF1.

A necessary adaptation to intermittent rather than continuous feeding is the storage of energy as adipose tissue. In turn, adipose tissue secretes adipokines that regulate appetite. We found that TG^MAT^ pups had a delay in their ability to acquire abdominal WAT stores and consequently manifested serum hypertriglyceridemia and TAG storage in the liver and muscle. In addition, serum leptin levels were reduced. *Dlk1* overexpression inhibits differentiation of white adipocytes in vitro and in vivo (Sul, 2009), an effect that is thought to be mediated by circulating DLK1. We found that delayed WAT differentiation was associated with elevated serum DLK1.

The requirement for nonshivering thermogenesis in the perinatal period of rodents is well documented and its regulation understood (summarized in Cannon and Nedergaard, 2004). However, adequate BAT recruitment is also necessary to support thermogenesis in small animals that are newly independent of the nest. Our work highlights this second period of thermogenic vulnerability because misexpression of chromosome 12 imprinted genes results in compromised BAT recruitment and maturation, ultimately leading to a failure to maintain body temperature during the third postnatal week.

In primary BAT cultures, *Dlk1* expression correlates with the undifferentiated state (Hernandez et al., 2007). We show that *Dlk1* is dynamically regulated during BAT development in vivo and that maintenance of elevated *Dlk1* expression during a critical developmental window retards differentiation. During embryonic development, BAT arises from a *Myf5*-positive population of the dermomyotome that differentiates into adipocytes following the expression of lineage marker *Prdm16* (Seale et al., 2009). *Dlk1* is expressed in the dermomyotome and in the stromal population of cells surrounding the BAT from its appearance at e15 ([Yevtodiyenko and Schmidt, 2006] and Figure S1). In TG^MAT^ mice, elevated *Dlk1* precedes alteration in the expression of *Prdm16* (Figure 4H). Thus, we suggest that *Dlk1* is upstream of *Prdm16* in the BAT differentiation program. PRDM16 function is required during adipogenic differentiation to promote BAT-cell character and it acts with PPARγ to increase the sensitivity of cells to β-adrenergic signaling (Seale et al., 2009). Consistent with this, we found that β-adrenergic signaling was compromised in the mutants because they failed to upregulate the expression of β3 adrenoreceptors prior to weaning.

A crucial step in the control of thermogenesis is the transcriptional activation of UCP1. *Cis*-acting factors in the upstream region of the gene act to integrate sympathetic stimulation with other cellular signaling pathways to achieve maximal mRNA expression (summarized in Collins et al., 2004). T3 elevates the expression of *Ucp1* via a thyroid hormone response element in the 5′ region of the *Ucp1* gene (Bianco and Silva, 1987). In addition, BAT must be in a terminally differentiated state, with available triglyceride fuel, to achieve maximal *Ucp1* expression; this is achieved by the inclusion of a site for PPARγ, a nuclear receptor required for differentiation of adipocytes, in the upstream region of the gene (Rabelo et al., 1997). Therefore, in our model local overexpression of *Dlk1*, together with reduced TH signaling, combine to generate BAT that is unable to mount a sufficient cold response.

Imprinted genes are emerging as key regulators of postnatal metabolic processes, and a number of imprinted genes have effects on essential metabolic/physiological adaptations (Charalambous et al., 2007). The kinship theory of the evolution of imprinting predicts that paternally expressed genes should act to reduce thermogenic output of individuals in a litter of mixed paternity (Haig, 2004). Our data is broadly consistent with this prediction but, surprisingly, we saw no impairment in thermogenesis during the nest phase (P0–P11); later, animals were unable to maintain *Ucp1* expression during the transitional phase to independent movement and feeding.

Studies of genetic variation associated with human disease are revealing that most variation occurs within intergenic regulatory regions rather than coding sequences (Mattick, 2009). Although the model we describe here was generated by genetic manipulation, it illustrates how a regulatory mutation influencing gene dosage can have a dramatic effect on whole-body physiology. The regulatory mutation we describe does not cause a complete loss of imprinted gene function but rather alters gene dosage and, thus, supports a model in which epigenetic modulation of this important class of genes might underlie the range of mammalian variation in metabolic rate and energetic set points observed within and outside the normal range.

## Experimental Procedures

### Breeding of Transgenic Animals

The Gtl2LacZ insertion and PCR genotyping were performed as described previously (Schuster-Gossler et al., 1996). Because genetic background has been reported to modify the action of this transgene (Steshina et al., 2006), we backcrossed Gtl2LacZ mice for multiple generations with 129Sv stock before commencing this study. TG^MAT^ animals were generated from either TG^MAT^ or TG^PAT^ females crossed with 129Sv (WT) males. We observed no significant differences in embryonic growth or postnatal phenotype between the two grand maternal types, so the data is combined. The day of vaginal plug was considered day E1. Since mutant animals were growth-retarded, we delayed the weaning of all litters until the fourth postnatal week (P28–P30). Animals were housed at a density of 3–4 per cage in a temperature-controlled room (20–22°C) with a 12 hr light/dark cycle, except for thermoneutrality experiments, when cages were placed on a hot pad at 30°C. Cold-challenged animals were removed from the nest and separated at room temperature (21.5°C) in the presence of wet-mash food for 2 hr prior to measurement of rectal temperature at a depth of 5 mm with a TES-319 digital thermometer (TES Electronic Corp). All experiments involving mice were carried out in accordance with UK Government Home Office licensing procedures.

### Expression Studies

Total RNA was prepared from snap frozen tissues using Trizol (Invitrogen) according to the manufacturer's instructions. mRNA was extracted from100 μg of the total RNA Dynalbeads Oligo (dT)_25_ kit (Invitrogen) following the supplied protocol, and we used 0.5 μg mRNA per sample in a standard northern blotting protocol with probes complementary to *Dlk1* and *Gapdh*, described previously (da Rocha et al., 2009). RNase protection assays were performed essentially according to Isaacs, 1992 (Isaacs et al., 1992). P32 labeled probes were generated by in vitro transcription from cloned fragments of *Rtl1* and *Rtl1AS* (between nucleotides 2444–2835, NM184109), *Dio3* (571–776, NM172119), *Dlk1* (1304–1570, BC052157), and alpha tubulin (178–277, BC056169) using T7 (Promega) and SP6 polymerases (Ambion). Yeast total RNA was used as a negative control and did not generate a signal of the protected size with any of the probes. For quantitative real-time PCR (qRT-PCR), cDNA was generated from 2 μg total RNA which had been treated with DNase I (Promega), using the RevertAid H Minus cDNA Synthesis Kit (Fermentas) with random primers. A SYBR Green assay was performed with SensiMix (Quantace) using the primers in Table S4. Quantification was performed using the relative standard curve method, and target gene expression was normalized to the expression of *Hprt*, the expression of which did not differ between the groups (data not shown). All primers amplified with efficiency greater than or equal to 85%.

### Immunohistochemistry and Proliferation

Rehydrated wax sections were incubated with an anti-DLK1 antibody (abcam 1:100 for embryo and Enzo 1:100 for BAT), UCP1 (abcam 1:200), or Ki67 (DAKO 1:300) according to published methods (da Rocha et al., 2009). Determination of Ki67-positive cell fraction was carried out by counting positive cells in 10 randomly chosen fields from 4 independent sections per animal.

### Serum Biochemistry

Serum peptides were quantified by ELISA: insulin (Mouse Ultrasensitive Insulin ELISA Kit, Crystal Chem), leptin (Crystalchem), and IGF1 (Rat/Mouse IGF1 ELISA, IDS), free triiodothyronine and total triiodothyronine (fT3 and tT3, Alpha Diagnostic International), thyroxine, and thyroid stimulating hormone (T4 and TSH, American Research Products, Inc.), all according to the manufacturer's instructions. A sandwich DLK1 ELISA was devised utilizing primary antibodies from ENZO (1:100) and R&D Systems (1:200) as well as HRP-conjugated secondary antibodies (1:500, DAKO) and TMB detection. Serum sample values were obtained by comparison to a standard curve of recombinant DLK1 (R&D Systems). Serum and tissue triglycerides were measured using the Sigma Triglyceride Determination Kit according to (Norris et al., 2003).

### D3 Assays

D3 activity was determined as described (Hernandez et al., 2006). A suitable volume of tissue homogenate was used in the enzymatic reaction to ensure that deiodination did not exceed 20% and was proportional to the amount of protein content.

### BAT Oxygen Consumption

Oxygen consumption rates were measured in BAT explants (∼20 mg) maintained in Krebs-Henseleit buffer at 37°C, using Clark-type oxygen electrodes (Strathkelvin Instruments, Strathkelvin, UK). Rates were measured in the presence of NE (1 μM) and succinate (10 mM), final concentration, to stimulate electron transport at the mitochondrial inner membrane. Subsequent addition of the electron transport chain inhibitors antimycin A (5 μM) and sodium azide (10 mM) confirmed that differences in oxygen consumption rates were of mitochondrial origin (data not shown).

### Statistical Analysis

All statistical tests were performed using the GraphPad Prism Software Version 4.00 for Windows (GraphPad Software, San Diego California USA, www.graphpad.com). Specific tests, significance values, and number of samples analyzed are indicated in the respective figure and table legends. Figures display the mean and error bars represent the standard error of the mean.
