# Supplementary material for: Imprinted Gene Dosage Is Critical for the Transition to Independent Life
Source: Cell Metab. 2012 Feb 8;15(2):209–21. doi: 10.1016/j.cmet.2012.01.006 (PMC3314949; doi:10.1016/j.cmet.2012.01.006)
Supplement: Document S1. Figures S1–S4 and Tables S1–S3 [file mmc1.pdf]

Cell Metabolism, ***Volume 15***

Supplemental Information

Imprinted Gene Dosage Is Critical for the Transition to Independent Life

Marika Charalambous, Sacramento R. Ferron, Simao T. da Rocha, Andrew J. Murray, Timothy Rowland, Mitsuteru Ito, Karin Schuster-Gossler, Arturo Hernandez, and Anne C. Ferguson-Smith

Supplementary Figure 1

A

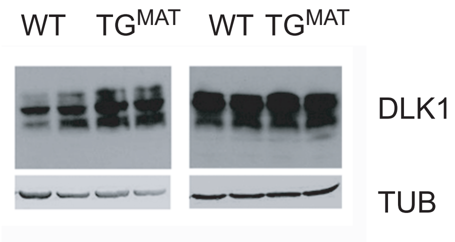

B

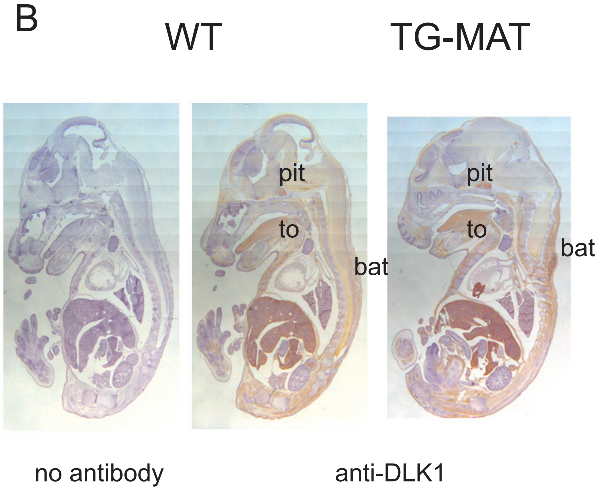

C

	WT mean	WT s.e	TG-MAT mean	TG-MAT s.e	*p*	litters
**e16**						
number in litter	3.7	0.3	3.1	0.2	*ns*	12
% in litter	54	3	46	3		
embryo weight mg	404	11	415	8	*ns*	
placenta weight mg	76	2	84	3	*<0.01*	
**e17**						
number in litter	3.0	0.9	2.6	0.7	*ns*	5
% in litter	44	12	56	12		
embryo weight mg	646	10	663	25	*ns*	
placenta weight mg	77	2	94	4	*<0.05*	

Figure S1. Expression of *Dlk1* in e16 Conceptuses, Related to Figure 1

(A) DLK1 expression in the embryo and placenta assayed by Western blotting, alpha tubulin (TUB) was used a s a loading control.

(B) DLK1 spatial expression in the WT and TG^{MAT} embryo assayed by anti-DLK1 immunohistochemistry, note abundant expression in the developing pituitary (pit), tongue muscle (to) and brown adipose tissue (bat).

(C) Wet weights of e16 and e17 TG^{MAT} embryo and placenta and their WT littermates, with average number and proportion of each genotype within the litter.

Figure S2

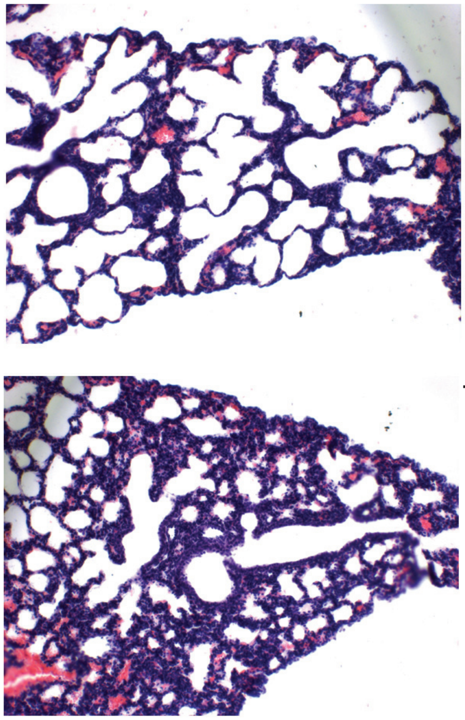

Figure S2. Lung Immaturity in Perinatal TG^{MAT} Animals, Related to Figure 2

H&E sections of WT (top) and TG^{MAT} lungs at P0 at 40x. Note alveolar sacs appear smaller and thicker in the mutant.

Supplementary Figure 3

A

	WT			TG-MAT			
	mean	se	n	mean	se	n	p
**P6**							
serum TAG mg/L	2540	138	20	2370	118	23	ns
liver TAG mg/g	208	40	4	474	115	7	ns
muscle TAG mg/g	2723	161	4	2996	162	7	ns
serum DLK1 ug/mL	231	8	8	242	6	12	ns
**P11**							
serum TAG mg/L	1470	136	17	2080	144	8	<0.01**
liver TAG mg/g	264	31	5	317	22	5	p<0.05*
muscle TAG mg/g	1944	140	12	2380	301	5	ns
WAT TAG mg/g	4325	95	4	3800	100	4	p<0.05*
serum DLK1 ug/mL	191	8	12	225	14	7	<0.05*
**P14**							
serum TAG mg/L	755	73	13	516	54	6	<0.05*
liver TAG mg/g	83	3	11	101	7	6	<0.01**
muscle TAG mg/g	1542	301	4	2365	288	5	<0.05*
WAT TAG mg/g	5475	245	4	4700	200	4	<0.05*
serum DLK1 ug/mL	81	2	10	96	2	6	<0.05*
**P21**							
liver TAG mg/L	56	4	14	54	4	14	ns
serum TAG mg/L	915	96	14	984	29	6	ns
serum DLK1 ug/mL	45	2	10	54	4	6	<0.05*

B

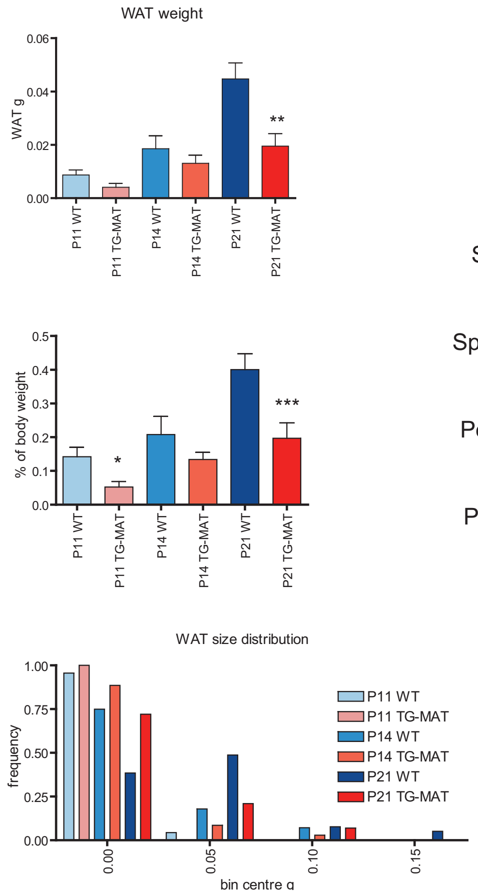

C

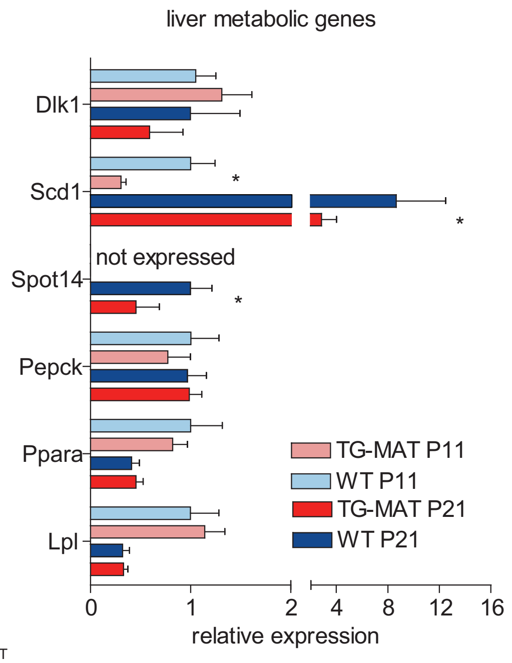

Figure S3. Transition from a High Fat to a High Carbohydrate Diet Is Impaired in TG^{MAT} Animals, Related to Figure 2

(A) Serum and tissue triglyceride at P6, P11, P14 and P21, values as mg/L triglyceride triolein equivalents (serum) and mg triolene equivalents/g tissue. Serum DLK1 at P6, P11, P14 and P21 ug/mL.

(B) WAT weight at P11-P21 as total weight (top), weight normalised to body weight (middle), and a frequency distribution with binned WAT weight on the x axis. P11 n = 27 WT, 16 TG^{MAT} from 6 litters, P14 n = 37 WT, 20 TG^{MAT} from 5 litters and P21 43 WT and 35 TG^{MAT} from 9 litters. Black bars WT and grey bars TG-MAT mean weight/mean normalised weight +/- se, *p<0.05, **p<0.01, ***p<0.001 by Mann-Whitney U test between genotypes at each time point.

(C) Real time quantitative PCR analysis of pre-weaning liver gene expression. as described in Figure 3D, except all values are represented as relative to WT at P11 = 1, as both time points were analysed during the same experiment. Error bars show S. E. M.

Supplementary Figure 4

A. Juvenile D3 activity

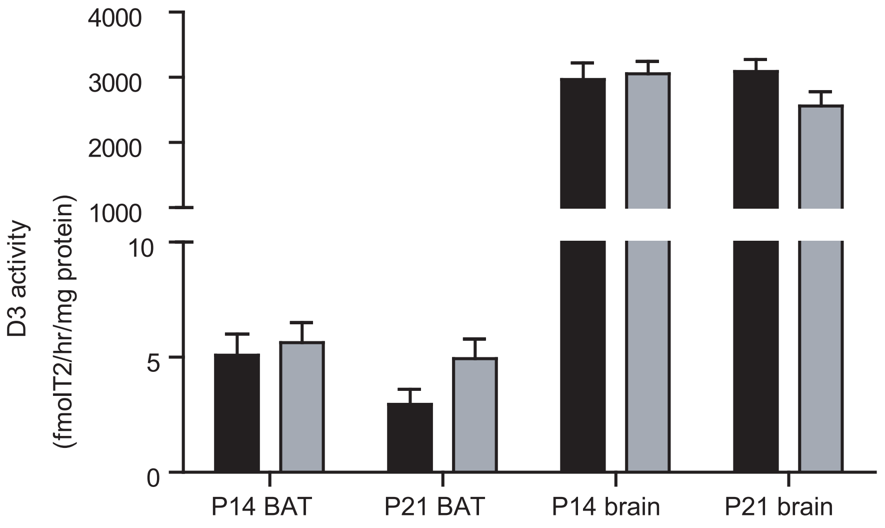

B. P21 gut morphology

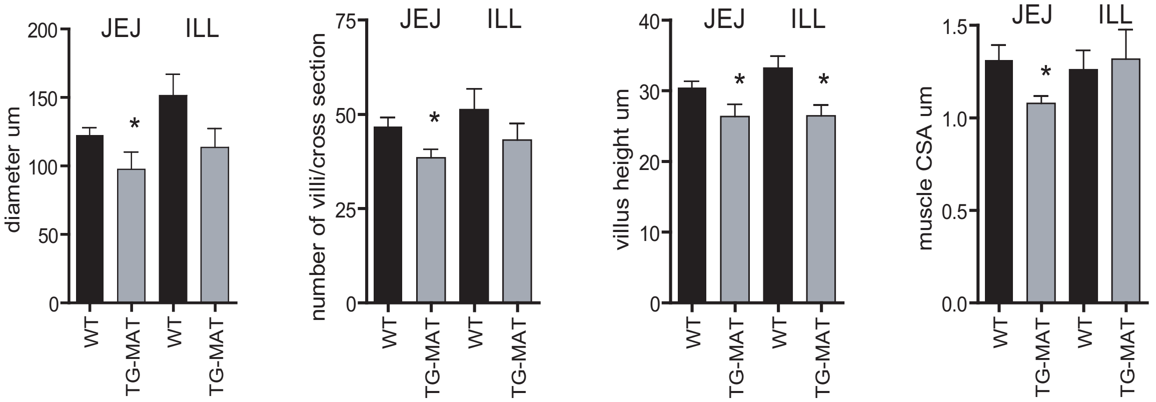

C. Circulating IGF1

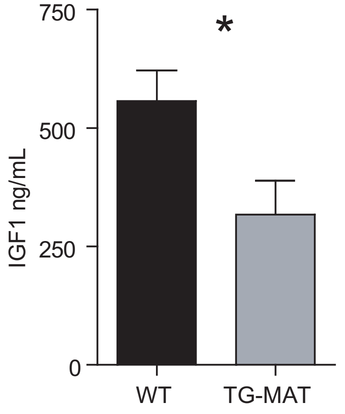

Figure S4. Thyroid Hormone-Associated Parameters, Related to Figure 3

(A) D3 enzyme activity assays performed on brain and BAT from mutant and WT littermates at P14 and P21. Genotypes were compared for each tissue and no significant differences were found (Mann-Whitney U test, n = 3-9 individuals/genotype).

(B) Morphometry of the jejunum and ileum of WT (n = 8) and TGMAT (n = 6) animals at P21. Images of 4 haemotoxylin and eosin sections from each animal were measured.

(C) Circulating IGF1 at P21 (WT n = 8, TG^{MAT} n = 7, *$p<0.05$ Mann-Whitney U test). Error bars show S. E. M.

Table S1. Early Life Adipose Tissue Weight and Serum Parameters

	WT		TG-MAT			
	mean	*s.e.m*	*mean*	*s.e.m*	*p*	*litters*
**P0**						
**total weight g**	1.72	*0.05*	1.59	*0.10*	ns	
**glucose mg/dL**	3.9	*0.4*	3.9	*0.3*	ns	
**BAT g**	0.017	*0.002*	0.015	*0.001*	ns	
**BAT%**	0.922	*0.094*	0.750	*0.030*	ns	
**n**	10		7			4
**P3**						
**total weight g**	2.28	*0.14*	2.24	*0.15*	ns	
**glucose mg/dL**	4.5	*0.4*	4.9	*0.9*	ns	
**BAT g**	0.020	*0.002*	0.021	*0.003*	ns	
**BAT%**	0.866	*0.044*	0.938	*0.077*	ns	
**n**	9		6			2
**P6**						
**total weight g**	4.08	*0.09*	4.33	*0.13*	ns	
**glucose mg/dL**	7.0	*0.4*	9.1	*0.8*	0.009**	
**BAT g**	0.030	*0.001*	0.037	*0.004*	0.044*	
**BAT%**	0.075	*0.031*	0.853	*0.084*	ns	
**n**	25		11			4
**P11**						
**total weight g**	6.65	*0.18*	6.67	*0.16*	ns	
**glucose mg/dL**	6.5	*0.4*	6.3	*0.3*	ns	
**BAT g**	0.040	*0.002*	0.036	*0.002*	ns	
**BAT%**	0.592	*0.018*	0.565	*0.025*	ns	
**n**	27		16			6
**P14**						
**total weight g**	7.84	*0.20*	7.06	*0.22*	0.012*	
**glucose mg/dL**	8.3	*0.3*	6.9	*0.4*	0.016*	
**leptin mg/dL**	6.1	*1.3*	3.5	*1.2*	ns	
**insulin mg/dL**	0.30	*0.10*	0.20	*0.07*	ns	
**BAT g**	0.088	*0.003*	0.070	*0.003*	<0.0001***	
**BAT%**	1.130	*0.025*	1.010	*0.029*	0.003**	
**n**	37		20			5
**P21**						
**total weight g**	10.63	*0.29*	7.91	*0.30*	<0.0001***	
**glucose mg/dL**	8.6	*0.3*	7.1	*0.5*	0.01**	
**leptin mg/dL**	3.7	*0.4*	1.5	*0.3*	<0.05*	
**insulin mg/dL**	0.813	0.173	1.72	0.827	ns	
**BAT g**	0.090	*0.005*	0.079	*0.005*	ns	
**BAT%**	0.832	*0.030*	1.030	*0.064*	0.008**	
**n**	43		35			9

Summary of weight data collected from animals sacrificed between P0 and P21. Serum glucose was measured from animals prior to death, and leptin and insulin measured at P14 and P21. Interscapular BAT weights are represented as totals as well as a % of total body weight. Number of animals studied (n) for each time point is indicated, as well as number of litters examined. P values are the result of Student's t tests.

Table S2. Early Life Organ Weight

	WT		TG[-MAT]			
	mean	*s.e.m*	*mean*	*s.e.m*	*p*	*litters*
**P0**						
**brain g**	0.107	*0.002*	0.110	*0.001*	ns	
**brain %**	5.900	*0.134*	5.680	*0.005*	ns	
**n**	10		7			4
**P3**						
**brain g**	0.125	0.004	0.131	0.005	ns	
**brain %**	6.810	*0.012*	6.560	*0.295*	ns	
**n**	9		6			2
**P6**						
**stomach g**	0.080	*0.012*	0.094	*0.018*	ns	
**stomach %**	0.019	*0.003*	0.020	*0.004*	ns	
**liver g**	0.094	*0.004*	0.104	*0.007*	ns	
**liver %**	2.310	*0.071*	2.430	*0.149*	ns	
**brain g**	0.267	0.005	0.245	0.00555	0.02*	
**brain %**	6.720	*0.137*	5.710	*0.189*	0.0003***	
**n**	25		11			4
**P11**						
**stomach g**	0.087	*0.004*	0.094	*0.017*	ns	
**stomach %**	0.012	*0.003*	0.015	*0.001*	ns	
**liver g**	0.209	*0.018*	0.172	*0.009*	ns	
**liver %**	2.850	*0.123*	2.620	*0.089*	ns	
**brain g**	0.245	*0.006*	0.393	*0.014*	0.0057**	
**brain %**	5.580	*0.226*	5.360	*0.139*	ns	
**n**	16		11			4
**P14**						
**stomach g**	0.090	*0.006*	0.093	*0.008*	ns	
**stomach %**	0.013	*0.001*	0.013	*0.001*	ns	
**liver g**	0.267	0.008	0.219	0.010	0.0003***	
**liver %**	3.380	*0.045*	3.060	*0.070*	0.0001***	
**brain g**	0.413	*0.005*	0.365	*0.008*	<0.0001***	
**brain %**	4.820	*0.178*	5.490	*0.173*	0.015*	
**n**	37		20			5
**P21**						
**stomach g**	0.179	*0.008*	0.184	*0.016*	ns	
**stomach %**	0.019	*0.002*	0.020	*0.003*	ns	
**liver g**	0.420	*0.017*	0.293	*0.015*	0.0001***	
**liver %**	3.910	*0.087*	3.680	*0.146*	0.007**	
**brain g**	0.423	*0.003*	0.387	*0.005*	<0.0001***	
**brain %**	4.060	*0.094*	5.010	*0.134*	<0.0001***	
**n**	43		35			9

Summary of weight data collected from animals sacrificed between P0 and P21. Tissue weights are represented as totals as well as a % of total body weight. Number of animals studied (n) for each time point is indicated, as well as number of litters examined. P values are the result of Student's t tests.

Table S3. Primers Used in Real-Time Quantitative PCR Analyses

primer	F	R
Dlk1	GAAAGGACTGCCAGCACAAG	CACAGAAGTTGCCTGAGAAGC
Gtl2	GGACACACGGACACAGACA	TGTCCCACAGGAAATGTGCAA
Meg9/Mirg	GGACATCCATGGGAGAGCTA	AGAGCAGAAACCCCTCCTTC
Dio3	GCGACGTTGACTTCCTTATCATCT	ATGACGTAGAGGCGCTCAAAATAG
Prdm16	CAGCACGGTGAAGCCATTC	GCGTGCATCCGCTTGTG
HPRT	CAGGCCAGACTTTGTTGGAT	TTGCGCTCATCTTAGGCTTT
β1-AR		
β3a-AR		
β3b-AR	Cikos, 2005	
α1a-AR	GGTTCCCAAAGGAAACCTGT	GGTTTCATACCAGGGTGGTG
Ucp1		
Ucp3		
Ppara		
Scd1		
Lpl		
Pepck		
Pgc1a		
Dio2		
Pparg2	Lelliot, 2006.	
TSHb	Zhu, 2006.	
